# Supplementary material for: Effect of different designs of interspinous process devices on the instrumented and adjacent levels after double-level lumbar decompression surgery: A finite element analysis
Source: PLoS One. 2020 Dec 30;15(12):e0244571. doi: 10.1371/journal.pone.0244571 (PMC7773253; doi:10.1371/journal.pone.0244571)
Supplement: Attachment — Submitted filename: Response To Reviewers.docx [file pone.0244571.s001.docx]

**Response to reviewers:**

Reviewer #1: The authors present a finite element study on 3 interspinous devices and analyse the range of motion, stress on the disc and facet joints as well as contact forces on the spinous processes to investigate the causes of spinal process fractures.
The authors could find that the devices have different Impacts on the spinal structures, especially in 2-segment Implantation.

I have several issues:
- The M-PEEK was developed in the institution of the authors. Please state the connection to the developer of the device.

**Response: The first author, corresponding author and the developer (Dr. Hsin-Chang Chen) of M-PEEK device were colleagues in Department of Biomedical Engineering, National Yang-Ming University, Taipei, Taiwan. We study the same field of spinal biomechanics.**

- The function and validity of the ANSYS Software should be explained in more detail (at least 2-3 sentences), please do not refer to other studies only.

**Response: The statements of the model validation have been added in Lines 100-105 as follows, *“The intact model was validated by applying a pure moment of 7.5 Nm in flexion, extension, torsion, and lateral bending, respectively; all degrees of freedom at the inferior surfaces of the L5 vertebra were constrained. The ROM of each vertebra and the facet joint forces at all segments was computed. Subsequently, 1000 N axial compressive force was acting on the top of L1 vertebra and the intradiscal pressures were calculated in comparing to published literatures [16-18].”***

Reviewer #2: General comments
. Meaning of any abbreviation must be mentioned on its first use in the abstract (as a separate entity) and in the manuscript text. Afterword, the abbreviation must be used instead of the original word. The term interspinous devices was used throughout the introduction and then the abbreviation IPD was used from line 80. Lumbar spinal stenosis was abbreviated as (LSS) in line 40 and then the full term (lumbar spinal stenosis) was used in the "Discussion" section in lines 217-218.
For any term used one time, no need to be abbreviated e.g Intermittent neurogenic claudication (INC) (line 48)

**Response: The abbreviation problems of IPD, LSS and INC have been fixed in revised manuscript.**

.Figures, figure legend and tables must be moved after the end of the text. Then figures and tables are cited in the text. Examples: lines 120-122: “Fig 1 Finite element model of the a) intact lumbar spine implanted with two interspinous spacers of (b) DIAM, (c) pedicle screw-based M-rod (M-PEEK) system and (d) Coflex at L2/L3 and L3/L4 segments”. Lines 172-173 & “Fig 2. Difference in ROM as a percentage of the intact model (% of intact) at the implanted and adjacent levels in flexion, extension, axial rotation, and lateral bending” must be moved to Figure legend after the references

**Response: The figure legend and tables have been moved after the end of the text in the revised manuscript.**

Abstract
Lines 22-23; "Recently, various designs and material manufactured interspinous process devices (IPDs) 23 are on the market in managing symptomatic lumbar spinal pathology" Lumbar spinal pathology should be changed to "Lumbar spinal stenosis".

**Response: The term “Lumbar spinal pathology” has been changed to “Lumbar spinal stenosis” in Line 23 in our revised manuscript.**

Introduction
Is too long with a lot of repetitions. It must be shortened and concentrate on the aim of the current study.

**Response: Thank you for review’s suggestion. The introduction section has been shortened and concentrate on the aim of the current study as our revised manuscript.**

Line 85: “..middle spinous process...” what does it mean? Middle in relation to what?

**Response: The “..middle spinous process...” means “the intervening spinous process in patients with adjacent, double-level IPDs”. This term has been rewriting as “the intervening spinous process in patients with adjacent, double-level IPDs” in Line 80 and Line 251.**

Results
The authors did not perform any statistical testing for their results

**Response: The results of ROM or contact force in the specific segmentation or disc of specific model in this study is unique and repeatable when the boundary condition, loading condition and material properties were the same. Therefore, we did not perform any statistical testing for our results.**

. Lines 177-178: “..for the Coflex model, the ROM was more variation as compared to the intact model, as depicted in Fig 2 and Table 2.” What is the meaning of this word?

**Response: This sentence means the ROM differences between the Coflex and INT models are larger and obviously in lateral bending. In contract, the ROM differences between the DIMA/M-PEEK and INT models are very similar.**

. Discussion is long and should be shortened

**Response: Thank you for review’s suggestion. The discussion section has been shortened and concentrate on the aim of the current study as our revised manuscript.**

. Line 237: “Effect“ is better to be used than “result”
**Response: The “but the result on other movements was not significant” has been modified as “but movements on other motions were not significant” in Line 198-199 of our revised manuscript.**

. Lines 316-317: “Also, the adjacent segments must compensate more for range of motion when using rigid implants than mobile devices [33] because the use of the hybrid method.” What is the meaning of this word?

**Response: With the use of the hybrid method, the moment placed on the fusion segment increases proportionally to the additional adjacent segment motion. Therefore, adjacent segments must compensate more when using rigid implants than mobile devices [33]. The stiffness of the adjacent segments directly impacts the motion distribution among these segments. Due to its nonlinear behavior, the spine offers low resistance to movement when in its neutral position, but gradually stiffens when loaded. This means that the stiff adjacent segments will typically have a lower range of motion than mobile segments. Therefore, even though all segments are subjected to the same loading, the mobility of adjacent segments may vary.**
