# Supplementary material for: Effect of different designs of interspinous process devices on the instrumented and adjacent levels after double-level lumbar decompression surgery: A finite element analysis
Source: PLoS One. 2020 Dec 30;15(12):e0244571. doi: 10.1371/journal.pone.0244571 (PMC7773253; doi:10.1371/journal.pone.0244571)
Supplement: Attachment — Submitted filename: Response to Reviewers.docx [file pone.0244571.s002.docx]

**Dear Editor and reviewers:**

*Thank you so much for your work in reviewing this manuscript and your comments are addressed below.*

**Response to reviewers:**

Reviewer #1: The authors have addressed all comments adequately.

*Thanks very much for your work in review this manuscript and we have made the changes/corrections you have suggested.*

Reviewer #2: General comments
English language is poor and must be improved.
*Thank you for these comments. It is good to learn when intended messages do not translate well to the reader due to the English written. we have had a professional scientific editor to proofread and polish this manuscript and adjusted the section on clustering*

Introduction

Lines 51-52: “The lumbar interspinous process device (IPD) is based on this principle and is placed at a symptomatic level to limit the extension of the lumbar spine” What do the authors mean by “this principle”?

*The “this principle” implies the “decompressive laminectomy with the dorsal decompression procedure” this is the common surgical principle, which addressed in the first paragraph of Introduction. We added “dorsal decompression” to more clarification, at Line 50 (clean MS)*

Repetitions are still present: authors reported mechanism of action of these IPDs at 3 sites in the Introduction only:

Lines 51-53: …and is placed at a symptomatic level to limit the extension of the lumbar spine and maintain a relatively flexible mobility at the intradiscal level to achieve the goal of symptom relief.

Lines 57-58; These IPDs are designed to enlarge the canal space and to relieve pain by offloading the entrapped spinal root nerves and as the intralaminar stabilizer.

Lines 59-62: Such devices provide significant improvements in managing the spinal pathology by rapid indirect decompression, and progressively decrease intradiscal and facet loads, which restores the neuroforaminal spaces and stabilize the spinal column in different postures.
*Thanks your comment, the three paragraphs were attempted to address the mechanism of IPD, it did sound redundant we have rewritten this paragraph and coincide the sentences as shown at Line 50 to 56*

Lines 71-72: What do the authors mean by “with limited disclosure information” ?

*The FDA approved the surgical procedure limited to two segments only, however, some surgeons might have over two-level implication without revealing in available literatures. We have deleted the words to avoid the confuse adding.*

Line 87: This study aimed to investigate the effect of different designs and material used of the IPDs in the management of neurological pain due to LSS” This study did not investigate any point related to effect of these IPD on neurological pain. The goal of the study was not clarified in the “Introduction” but clarified in the “Discussion” Lines 187-190 to be “The goal of this work was to look into alterations in the biomechanical characteristics of the lumbar spine after implanting an IPD across two adjacent levels and then to compare the significance with a single level instrumented model.” I would recommend that the authors clarify the aim of the work in the “Introduction”.

*Thanks, your valued suggestion, the aim of the work had been moved from Discussion section and clarified in the Introduction section in our revised manuscript. In addition, the effect of IPDs on neurological pain was also removed from the goal of this work.*

Material and Methods

Lines 188-119: the supraspinous and interspinous ligaments were preserved in both the DIAM and M-PEEK models. Do this make difference between the models that may affect the results? The authors must answer this question in the discussion.

*As indicated at Line 107,* due to design consideration and following real surgical procedures, *in the Coflex surgical procedure, the supraspinous and interspinous ligaments have to dissect in order to insert the implant.*

*As far as the contribution of the motion restrain of the ISL and SSL, the Young modulus in this study are 10 and 8 Mpa for ISL and SSL, respectively. Which is very small value in comparison with the cortical bone and implants. Literatures revealed that lumbar intervertebral disc and facet joints are the major load carrier during the functional unit motion. Our results revealed that this difference between Coflex and DIAM/M-PEEK affected the ROM in flexion. The ROM of instrumented levels in Coflex model in flexion was higher than DIAM model, removing the supraspinous and interspinous ligaments might be one of the factors, but the dimension and location of the implant as the pivot of extension could be the major cause. Nevertheless, it did not affect our main findings of adjacent level problems and a fracture of the spinous process. In addition, this is also a limitation in this study to have identical ligaments constraints in each model. Thank you for the comment and your inquiry was addressed in the discussion of this study, starting at the Line 257-265.*

Conclusions

“Of the three devices, the Coflex implant resulted in the most limited ROM in extension and highest annulus stress on adjacent level, which may result in accelerated degeneration of the adjacent segments as the sandwich phenomenon”. What is the relation between the accelerated degenerative process and the fracture of the spinous process of the middle vertebra on applying IPD at 2 adjacent level?

*So far there is no study reveals the relationship between degeneration of the adjacent segments and the sandwich phenomenon on spinous process. This statement had been rewritten as “Of the three devices, the Coflex implant shows the limited ROM at the instrumented level but compensating larger ROM at the two adject segments. In combined with highest annulus stress on adjacent level, which may accelerate the degeneration of the adjacent segments.” in Conclusion section in our revised manuscript. Staring line 275-277*

**Thank you again for all your comments. They have helped us develop the manuscript considerably.**
